# Spectroelectrochemical Studies on Quinacridone by Using Poly(vinyl alcohol) Coating as Protection Layer

**DOI:** 10.1002/cphc.201500165

**Published:** 2015-05-26

**Authors:** Sandra Enengl, Christina Enengl, Philipp Stadler, Helmut Neugebauer, Niyazi Serdar Sariciftci

**Affiliations:** [a] Linz Institute for Organic Solar Cells (LIOS), Physical Chemistry, Johannes Kepler University Linz Altenbergerstraße 69, 4040 Linz (Austria) E-mail: Sandra.Enengl@jku.at

**Keywords:** electrochemistry, IR spectroscopy, poly(vinyl alcohol), soluble materials, spectroelectrochemistry

## Abstract

Spectroscopic measurements in the infrared range combined with electrochemistry are a powerful technique for investigation of organic semiconductors to track changes during oxidation and reduction (p- and n-doping) processes. For these measurements it is important that the studied material, mostly deposited as a thin film on an internal reflection element, does not dissolve during this characterization. In this study we introduce a technique that allows infrared spectroelectrochemical characterization of films of these materials for the first time. In many cases so far this has been impossible, due to solubility in the oxidized and/or reduced form. This novel technique is shown on thin films of quinacridone by adding a protection layer of poly(vinyl alcohol) (PVA).

## 1. Introduction

Spectroelectrochemistry in the infrared range is a powerful tool that combines electrochemical studies with spectroscopic techniques. Herein, when a certain potential is applied while simultaneously spectroscopic measurements are recorded, we call this in situ spectroelectrochemical measurement throughout. In situ infrared spectroelectrochemistry has gained attention particularly in the field of organic semiconductors, especially on organic conjugated polymers.[1–3] These organic semiconductor materials are mostly deposited as thin films on a working electrode. Organic semiconductors exhibit highly interesting insulator-to-metal transitions from being insulators in their undoped, pristine state (with a band gap larger than 2 eV)[4] to being almost metallic upon doping.[5] This phenomenon gives rise to many applications, for example in organic optoelectronics.[6–8] Furthermore, such doping processes can be gate-field-induced in organic field-effect transistors (OFETs) for use in the organic electronics.[9]

**Figure 1:**
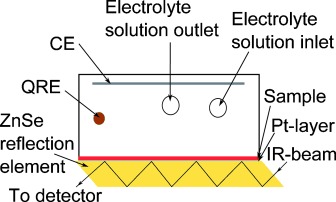
Experimental setup for in situ ATR-FTIR spectroelectrochemical measurements. We use a flow cell as electrochemical cell, where Pt serves as a counter electrode (CE) and a silver wire coated with AgCl as a quasi-reference electrode (QRE). As working electrode we use ZnSe crystal, sputtered with a thin layer of Pt, and covered with the studied material. The thin layer of Pt serves for contacting the reflection element. In ATR-FTIR spectroscopy the IR beam passes through the reflection element and the evanescent wave is used to probe the absorption of the thin film on the ZnSe crystal. For data registration, we apply a certain potential while simultaneously spectroscopic measurements are recorded.

Infrared spectroelectrochemistry can be used to observe and study electronic transitions in the mid-IR range as well as structural changes, which result in changes in vibrational absorption bands. For FTIR spectroelectrochemical measurements, where the incoming light interacts not only with the studied material but also with the used electrolyte solution, we avoid the problem of electrolyte absorption using the internal reflection mode as described in Ref. [10]. In attenuated total reflection (ATR) FTIR spectroscopy we pass the IR beam in an attenuated total reflection geometry and use the evanescent wave, which probes the absorption of a thin film sitting on the ATR crystal (Figure 1).[11] In this case the penetration depth into the sample is described due to an evanescent wave, which is typically between 0.5 and 2 µm. Since the penetration depth of the evanescent wave decreases exponentially, it is important that the studied material has good contact with the ATR crystal and that the material does not dissolve or lift off during characterization in their neutral, oxidized and reduced form in the electrolyte solution. Conjugated polymers, which are insoluble in the electrolyte solution, have successfully been characterized with such spectroelectrochemical measurements.[12–14]

In the last decades large-scale interest arose in the use of small molecular organic semiconductors, among them hydrogen-bonded pigments and dyes, in organic electronic devices.[15–18] OFETs and photo-diodes have already been applied successfully.[19, 20] The family of small molecular pigments and dyes has not yet been characterized in detail by in situ spectroelectrochemical measurements, since frequently the oxidized and reduced species dissolve during oxidation and reduction processes, respectively. A similar effect has already been discussed in Ref. [10], where the spectroelectrochemical response of fullerene films in the neutral and reduced form was influenced by solubility in organic solvents.

**Figure 2:**
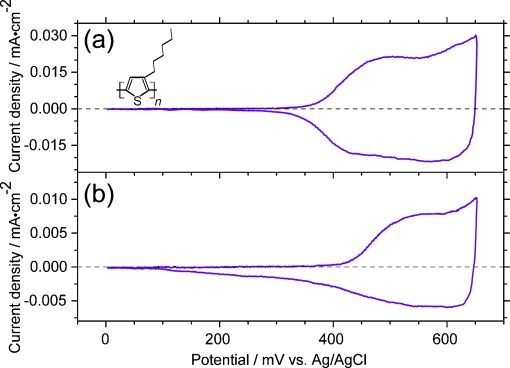
Cyclic voltammogram of P3HT a) without and b) with PVA are displayed. The inset shows the schematic chemical structure of P3HT.

Herein we present a method that allows the electrochemical and spectroelectrochemical measurement of such classes of molecules, which have been difficult to characterize so far. With this novel technique, the dissolution of the studied material in the oxidized or reduced form is prevented. This allows the detection of significant qualitative information on molecular level of the material, including basic characterizations in terms of electronic transitions and vibrational changes. These results are of high importance for applications in organic devices. Our technique is to add a thin and insoluble layer of PVA on top of the studied material, which prevents the dissolution of the oxidized and reduced form of the material as a binder matrix, but that allows the electrochemical reaction of the subjacent organic film. We demonstrate this possibility on the pigment quinacridone, a hydrogen-bonded organic semiconductor. We first show that a thin layer of PVA has no significant influence on the spectroelectrochemical measurement itself by testing this technique on the well-known organic conjugated polymer poly(3-hexylthiophene-2,5-diyl) (P3HT, structure shown as an inset in Figure 2 a). P3HT is insoluble in both the neutral and in the oxidized form in acetonitrile electrolyte solution, and spectroelectrochemical studies without a protection layer have already been carried out successfully.[21] The results with and without the PVA layer are compared. Then we show the successful application of PVA coating on the spectroelectrochemical characterization of a solid film of quinacridone (structure shown in an inset in Figure 4 a) in the infrared regime during oxidation, which is not possible without protection, due to dissolution of the film. The results show the formation of radical cations upon oxidation and enable one to see structural changes as observed due to the changes of the vibrational bands.

## 2. Results and Discussion

### 2.1. P3HT, a Reference Sample

As already mentioned before, P3HT has been successfully characterized by in situ spectroelectrochemical measurements.[21] (P3HT is just one representant for the class of organic conjugated polymers, where this kind of spectroscopic oxidation has been performed succesfully without any protection layer). For comparison, we test the influence of PVA on these measurements by investigating one P3HT sample with PVA and another one without PVA. In Figure 2 we show the cyclic voltammogram of P3HT without (a) and with (b) a layer of PVA. The oxidation of P3HT without PVA treatment, Figure 2 a, starts at 310 mV and has its maximum at 500 mV. In the case of the cyclic voltammogram of P3HT with the layer of PVA, presented in Figure 2 b, the oxidation starts at 360 mV and has its maximum at 550 mV. The results with and without the PVA layer are analogous, the whole oxidation reaction is not significantly influenced by using a layer of PVA on P3HT. The reaction between P3HT and the electrolyte solution is only moderately hindered.

**Figure 3:**
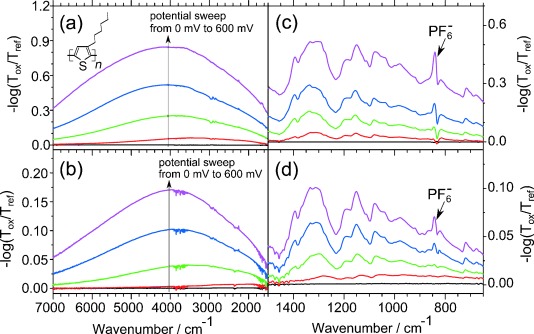
In situ differential spectra during oxidation of P3HT a) without and b) with PVA in the mid-IR range by changing the potential between 0 mV and 600 mV. The reference spectrum corresponds to the spectrum obtained at 0 mV and the arrow is indicating the potential change. The spectral range between 1500 cm^−1^ and 650 cm^−1^ during oxidation is shown for P3HT c) without and d) with PVA protection layer.

In Figure 3 we present the difference spectra in the mid-IR range recorded during the oxidation process of P3HT by sweeping the potential between 0 mV and 600 mV. The reference spectrum corresponds to the spectrum at 0 mV. Figures 3 a and 3 b show the spectra without and with the PVA protection, respectively. In Figure 3 a a broad absorption band in the mid-IR range, with a maximum at around 4000 cm^−1^ can be seen. In Figure 3 b the evolution of the same broad absorption band with PVA protection layer arises. By comparing Figures 3 a and 3 b, both difference spectra show an analogous electronic transition, but the absorbance is smaller for the measurement with PVA. The smaller signal in absorbance may be due to hindrance effects as well as the complex geometry for in situ ATR-FTIR measurements. In Figures 3 c and 3 d the spectral range between 1500 cm^−1^ and 650 cm^−1^ is shown without and with the protection layer, respectively. The peak at 843 cm^−1^ indicates that PF_6_^−^ counterions from the electrolyte solution enter and compensate the positive charges in the film. All other positive peaks show the appearance of IR-active vibrational (IRAV) modes. The assignment of these IRAV modes can be found in the Supporting Information. Again, in these two spectra the vibrational changes are analogous. PVA does not change the behavior of the spectra of P3HT, proving the applicability of the PVA protection method.

### 2.2. Quinacridone

**Figure 4:**
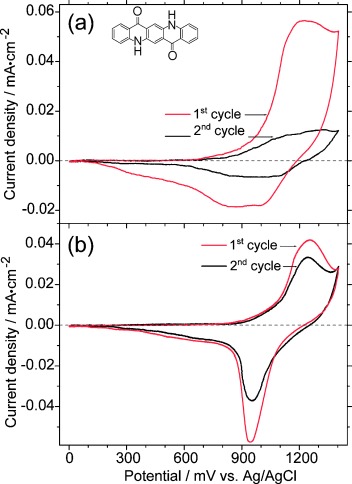
Cyclic voltammogram of quinacridone a) without and b) with PVA are displayed. The inset shows the schematic chemical structure of quinacridone. In both measurements the red curve corresponds to the first cycle and the black curve indicates the second cycle of the cyclic voltammogram.

Figure 4 a shows the cyclic voltammogram of a pristine quinacridone film. The oxidation for the first cycle, shown as a red curve, starts at 640 mV and has its maximum at around 1220 mV. The re-reduction shows a small broad peak centered at about 900 mV. Most of the material is dissolved during the oxidation process, indicated by the non-reversible cyclic voltammogram. The midpoint potential for the first cycle, defined as the average of the cathodic and anodic peak potentials, is around 1060 mV. With the small amount of quinacridone on the electrode left over from the first cycle, the second cycle, shown as a black curve, is much smaller, with ill-defined oxidation and re-reduction maxima. The problem that the material dissolves during oxidation, makes the spectroscopic characterization by ATR spectroelectrochemistry impossible.

In Figure 4 b we see the cyclic voltammogram of quinacridone with the usage of the protection layer PVA. In the first cycle, which is shown in red, the oxidation starts slightly delayed, at 770 mV compared to quinacridone without PVA and has its maximum at around 1260 mV. The re-reduction is now pronounced at around 950 mV and the midpoint potential is determined at 1100 mV. Despite the small shift compared to the value obtained for quinacridone without PVA the overall redox behavior looks similar, indicating that the influence of the PVA layer on electrochemistry is insignificant for spectroelectrochemical studies. Interestingly, the re-reduction peak in the first cycle without the PVA protection layer (Figure 4 a) is broader and more ill-defined compared to quinacridone with PVA. Probably the complex interplay between reduction of the still existing solid residual of the film and dissolved species are responsible for this effect. In the second cycle in Figure 4 a, shown as a black curve, the electrochemical response decreases to a very low value. Nearly no solid film material is left, which makes spectroelectrochemical measurements impossible. In contrast, with a PVA protection layer (Figure 4 b), the cyclovoltammetric response is similar to the first cycle (albeit somehow reduced), which shows a significantly higher reversibility of the system compared to the unprotected film. In this arrangement, a spectroelectrochemical characterization of quinacridone is possible, as described in the next section. It has to be mentioned that, at present, an extensive electrochemical study with detailed determination of electrochemical parameters like electron transfer rates is hindered by the complexity of the electrochemical ATR-FTIR setup. Such investigations will be the subject of further studies in the future.

**Figure 5:**
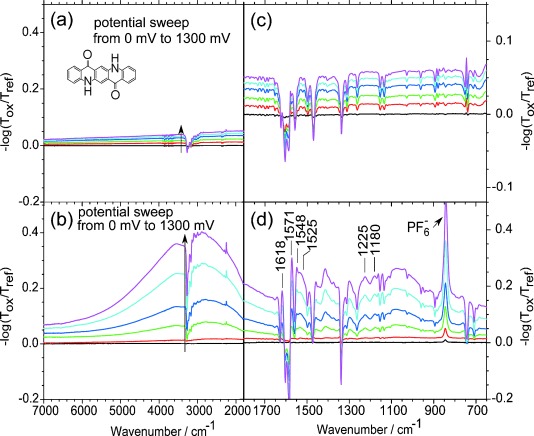
In situ differential spectra during oxidation of quinacridone a) without and b) with PVA in the mid-IR range are shown by changing the potential between 0 mV and 1300 mV. The reference spectrum corresponds to the spectrum obtained at 0 mV and the arrow is indicating the potential change. The spectral range between 1800 cm^−1^ and 650 cm^−1^ during oxidation is shown for quinacridone c) without and d) with PVA protection layer.

Spectroelectrochemical changes in the mid-IR range of quinacridone without PVA protection, during a potential sweep from 0 mV to 1300 mV with 0 mV taken as reference spectrum, are shown in Figure 5 a. The wavenumber range from 1800 cm^−1^ to 650 cm^−1^ is shown in Figure 5 c. In these two graphs a slightly shifting baseline and negative peaks, which correspond to the vibrations of pristine quinacridone, are discernable, indicating the dissolution of quinacridone during oxidation. The infrared spectrum of pristine quinacridone film and the characteristic vibrations are shown in the Supporting Information. No increasing absorption bands occur, again due to the dissolution of quinacridone during oxidation. Without the protection of this thin film, in situ spectroscopic measurements do not lead to significant results. The complete dissolution of quinacridone in the first oxidation during this in situ spectroscopic measurement, which occurs only partially in cyclic voltammetry, is due to the different timescales in these measurements. In cyclic voltammetry the oxidation process takes about two minutes, in spectroelectrochemistry around 14 min. Due to this longer measurement time, a complete dissolution occurs in the first oxidation process already.

**Scheme 1:**
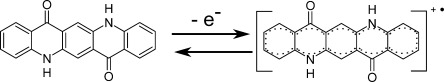
Chemical structure of quinacridone during oxidation.

If a thin layer of PVA on top of quinacridone protects the film during the measurement from dissolving, spectroelectrochemical studies become possible. Figure 5 b shows differential spectra during sweeping the potential between 0 mV and 1300 mV with the reference spectrum at 0 mV. A broad absorption band at 2890 cm^−1^ arises, which cannot be detected without the protection layer. In Figure 5 d the spectral range between 1800 cm^−1^ and 650 cm^−1^ is plotted, showing positive bands besides the already mentioned negative bands. At 1618 cm^−1^ and 1525 cm^−1^ new peaks appear. These peaks correspond to C=N stretching vibrations. Additionally, new C=C–H vibrations are observed at 1571 cm^−1^ and 1548 cm^−1^ and the peaks at 1225 cm^−1^ and 1180 cm^−1^ correspond to C–H deformation vibrations. The spectral changes may be an indication for the formation of semiquinoid structures (Scheme 1) similar to units described in the polaron lattice form of polyaniline.[22] The positive peak at 840 cm^−1^ represents the PF_6_^−^ vibration from the counterion in the electrolyte salt. The negative vibrations correspond to the pristine quinacridone, indicating loss in absorption, because the molecular structure of quinacridone changes during oxidation forming a radical cation. All in all, the PVA protection allows one to measure electrochemistry and spectroelectrochemistry on pigments, which would be impossible without any further protecting treatment.

## 3. Conclusions

This work reports the successful spectroelectrochemical characterization of molecular organic semiconductors during their redox processes, which lead to soluble redox states. We introduce a PVA protection layer, which prevents the dissolution of materials, and allows in situ spectroelectrochemical studies. With the well known material P3HT we tested that the PVA protection layer has no significant influence on the spectroscopic data. The spectroelectrochemical characterization of quinacridone with soluble redox states shows radical cation formation with structural changes to the molecule, as seen in the vibrational absorption changes. A full spectral analysis of quinacridone using electron paramagnetic resonance (EPR), FTIR, UV/Vis, and so forth, in comparison to pentacene will be reported elsewhere.

## Experimental Section

### Material Processing and PVA Protection Layer Technique

P3HT (91–94 % regioregular, Rieke Metals) was dissolved in chlorobenzene (VWR chemicals) with a concentration of 0.15 mol l^−1^ and spin-coated (70–80 nm) on ZnSe/Pt crystal and glass/ITO (15 Ω sq^−1^, Xinyan), respectively. Quinacridone (TCI) was purified by repeated temperature gradient sublimation and vacuum-evaporated (90–100 nm) on both substrates mentioned before. Subsequently, a thin layer (≈40 nm) of PVA (99+ % hydrolyzed, Aldrich) solution, 7 mg PVA dissolved in 1 mL deionized water, was spin-coated (900 rpm; 3 seconds) onto the pigment and polymer, respectively, and finally dried under air. The thickness of the PVA layer was measured by a Dektak profilometer (Bruker). This treatment protects solid films from dissolving during electrochemical studies.

### Electrochemistry

Electrochemical experiments were performed using a JAISSLE Potentiostat–Galvanostat IMP 88 PC under nitrogen atmosphere in a glove box. Pt served as a counter electrode (CE) and a silver wire coated with AgCl as a quasi-reference electrode (QRE), see Ref. [23] for fabrication details. A glass/ITO electrode covered with a thin film of the studied material, which was deposited as described above, has been used as working electrode (WE). In the case of P3HT, we used a concentration of 0.3 mol l^−1^. A solution of 0.1 m tetrabutylammonium hexafluorophosphate (TBAPF_6_, ≥99 %, Fluka Analytical) in acetonitrile (Roth) was used as an electrolyte solution. The potential of the QRE was calibrated by a ferrocene/ferrocenium redox couple. The midpoint potential of the ferrocene/ferrocenium redox couple was found to be +353 mV vs. Ag/AgCl (for a description of non-aqueous reference potential determination see for example, Ref. [24]). The cyclic voltammograms were recorded at a scan rate of 10 mV s^−1^ by sweeping the potential between 0 mV and 650 mV for P3HT and between 0 mV and 1400 mV for quinacridone.

### Spectroelectrochemistry

All spectroscopic measurements have been performed with an IFS 66/S spectrometer (Bruker) using the ATR-FTIR technique. For the spectroelectrochemical cell, shown schematically in Figure 1, we used ZnSe/Pt/studied material as a WE, Pt electrode as a CE and an Ag/AgCl electrode as a QRE. ZnSe was covered with a thin layer of Pt (≈7 nm) serving as IR-transparent electrode for contacting the WE. We used the same electrolyte solution as mentioned above, which flows through the cell during the measurement.

We changed the potential for P3HT between 0 mV and 600 mV in steps of 100 mV and for quinacridone from 0 mV to 1300 mV, also in steps of 100 mV. We plotted the spectra as −log(*T*_ox_/*T*_ref_), where *T*_ref_ is the spectrum obtained at 0 mV for quinacridone and P3HT, respectively, and all other related spectra during oxidation are denoted as *T*_ox_.

## Supporting Information

As a service to our authors and readers, this journal provides supporting information supplied by the authors. Such materials are peer reviewed and may be re-organized for online delivery, but are not copy-edited or typeset. Technical support issues arising from supporting information (other than missing files) should be addressed to the authors.

miscellaneous_information
