# Supplementary material for: Spectroelectrochemical Studies on Quinacridone by Using Poly(vinyl alcohol) Coating as Protection Layer
Source: Chemphyschem. 2015 May 26;16(10):2206–10. doi: 10.1002/cphc.201500165 (PMC4529658; doi:10.1002/cphc.201500165)
Supplement: Supplementary file 1 — miscellaneous_information [file cphc0016-2206-sd1.pdf]

CHEMPHYSCHEM

Supporting Information

Spectroelectrochemical Studies on Quinacridone by Using Poly(vinyl alcohol) Coating as Protection Layer

Sandra Enengl,* Christina Enengl, Philipp Stadler, Helmut Neugebauer, and Niyazi Serdar Sariciftci[a]

cphc_201500165_sm_miscellaneous_information.pdf

1 Determination of vibrations

1.1 Raman and infrared measurements

The Raman spectrum has been recorded on a FT-Raman Bruker MultiRam spectrometer with a liquid N_2 cooled Ge detector and a Nd:YAG laser at 1064 nm. For P3HT as well as for quinacridone the measurements are done in the powder form.

For infrared spectra the same spectrometer as described in the main text has been used using ATR-FTIR technique. P3HT is spincoated on ZnSe crystal and quinacridone is evaporated on ZnSe crystal, as mentioned before.

For the determination of the assignments of the IR and Raman vibrations we used the Gaussian 09 program, where DFT calculations were done using B3LYP and 6-31G(d) as basis set [1].

1.2 Results and discussion

Figure 1 shows (a) the Raman spectrum of pristine powder and (b) shows the infrared spectrum of P3HT as a thin film. Table 1 represents the main characteristic Raman and IR bands of P3HT as well as the IRAVs obtained by electrochemical oxidation of P3HT in the main paper. By using the Gaussian 09 program, we are able to assign the Raman and infrared vibrations, which coincide with the vibrations mentioned in [2, 3]. For quinacridone an analogous study has been carried out. Figure 2 shows (a) the Raman spectrum of pristine powder and (b) shows the infrared spectrum of quinacridone as a thin film. Table 2 represents the main characteristic Raman and IR bands of quinacridone. By using the Gaussian 09 program, we are able to assign the Raman and infrared vibrations, which coincide with the vibrations mentioned in [4].

References

[1] M. J. Frisch, G. W. Trucks, H. B. Schlegel, G. E. Scuseria, M. A. Robb, J. R. Cheeseman, G. Scalmani, V. Barone, B. Mennucci, G. A. Petersson, H. Nakatsuji, M. Caricato, X. Li, H. P. Hratchian, A. F. Izmaylov, J. Bloino, G. Zheng, J. L. Sonnenberg, M. Hada, M. Ehara, K. Toyota, R. Fukuda, J. Hasegawa, M. Ishida, T. Nakajima, Y. Honda, O. Kitao, H. Nakai, T. Vreven, J. A. Montgomery, Jr., J. E. Peralta, F. Ogliaro, M. Bearpark, J. J. Heyd, E. Brothers, K. N. Kudin, V. N. Staroverov, R. Kobayashi, J. Normand, K. Raghavachari, A. Rendell, J. C. Burant, S. S. Iyengar, J. Tomasi, M. Cossi, N. Rega, J. M. Millam, M. Klene, J. E. Knox, J. B. Cross, V. Bakken, C. Adamo, J. Jaramillo, R. Gomperts, R. E. Stratmann, O. Yazyev, A. J. Austin, R. Cammi, C. Pomelli, J. W. Ochterski, R. L. Martin, K. Morokuma, V. G. Zakrzewski, G. A. Voth, P. Salvador, J. J. Dannenberg, S. Dapprich, A. D. Daniels, O. Farkas, J. B. Foresman, J. V. Ortiz, J. Cioslowski, and D. J. Fox. Gaussian 09. Gaussian Inc. Wallingford CT **2009**.

[2] X. Y. Chin, J. Yin, Z. Wang, M. Caironi, and C. Soci. *Sci. Rep.*, 4:3626, **2014**.

[3] Y. H. Kim, D. Spiegel, S. Hotta, and A. J. Heeger. *Phys. Rev. B*, 38:5490–5495, **1988**.

[4] E. del Puerto, C. Domingo, J. V. Garcia Ramos, and S. Sanchez-Cortes. *Langmuir*, 30:753, **2014**.

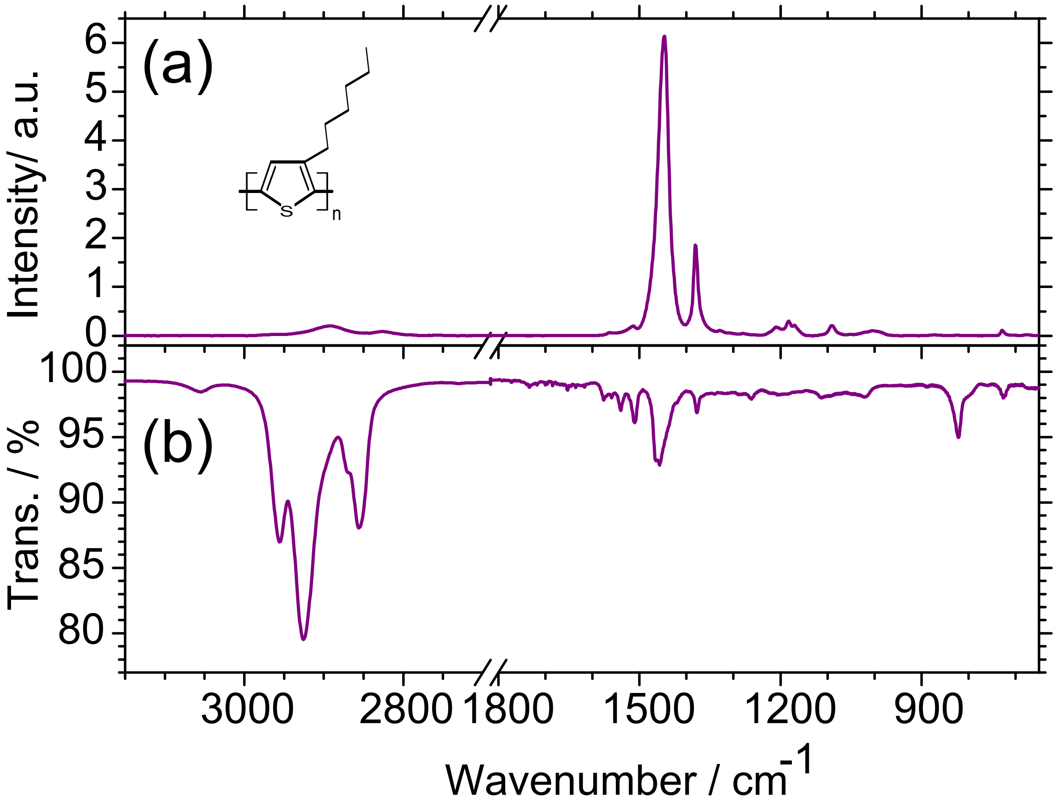

Figure 1: This shows (a) Raman spectrum of pristine powder and (b) ATR-FTIR spectrum of P3HT, deposited as a thin film and plottet in transmittance mode. The inset shows the schematic chemical structure of P3HT.

RAMAN-FREQUENCY	IRAVs	IR-FREQUENCY	ASSIGNMENT
2891		2926, 2955	ν CH_{arom}
2826		2856	ν CH
1447		1465 – 1576	ν $C{=}C_{arom}$
1381	1392		ν_s C-C
		1378	δ CH_3
	1320		
1208	1190		δ CH
1182	1151		δ CH
1091	1078		δ CH
1004	980		ν C-C
		821	δ_{op} CH
728	719		δ_{as} C-S-C
		726	δ CH of methylene group of hexyl chain

ν: stretching, δ: deformation, op: out of plane, s: symmetric, as: asymmetric

Table 1: Experimental Raman bands of powder, IRAVs upon electrochemical oxidation and IR bands of thin film of P3HT in wavenumbers (cm^{-1}) are described with the assignment of the vibrational spectra using the Gaussian 09 program.

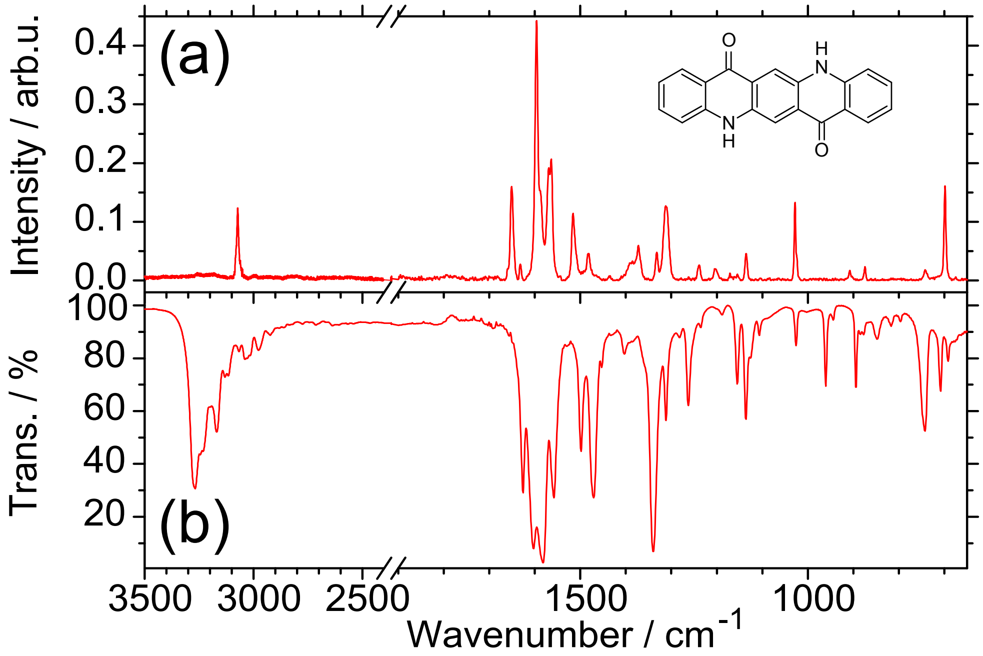

Figure 2: This shows (a) Raman spectrum of pristine powder and (b) ATR-FTIR spectrum of quinacridone, deposited as a thin film and plottet in transmittance mode. The inset shows the schematic chemical structure of quinacridone.

RAMAN-FREQUENCY	IR-FREQUENCY	ASSIGNMENT
	3267	ν_{s} NH
	3169	ν_{s} NH
3072		ν CH_{arom}
	2923 – 3130	ν CH_{arom}
1651		ν_{s} C=O
	1625	ν_{as} C=O
	1603	ν C=C, δ_{ip} NH
1596		ν C=C, ν C=O
	1582	ν C=C, δ_{ip} NH
1564		ν C=C
	1558	δ_{ip} NH, ν C=C
1516		ν C=C, δ_{ip} NH, δ_{ip} CH
	1498	ν C=C, δ_{ip} CH
	1470	ν C=C, δ_{ip} CH
	1339	ν C-C
1312		ν_{ip} CH, δ_{ip} NH
	1311	δ_{ip} NH, δ_{ip} CH
	1262	ν CN, δ_{ip} CH
	1135	δ_{ip} CH
1028		δ_{ip} C-C-C
	960	δ_{ip} C-C-C, δ_{ip} CH
	893	δ_{ip} C-C-C
	742	δ_{op} C-C-C
698		δ_{ip} C-C-C

ν: stretching, δ: deformation, ip: in plane, op: out of plane, s: symmetric, as: asymmetric

Table 2: Experimental Raman bands of powder and IR bands of thin film of quinacridone in wavenumbers (cm^{-1}) are described with the assignment of the vibrational spectra using the Gaussian 09 program.
